# A single-center experience of home parenteral nutrition for pediatric intestinal failure

**DOI:** 10.3389/fped.2025.1658512

**Published:** 2025-09-25

**Authors:** Honam Hwang, Dayoung Ko, Hee-Beom Yang, Joong Kee Youn, Jin Soo Moon, Jae Sung Ko, Hyun-Young Kim

**Affiliations:** ^1^ Department of Pediatric Surgery, Seoul National University Children’s Hospital, Seoul, Republic of Korea; ^2^ Department of Pediatric Surgery, Seoul National University Bundang Hospital, Seongnam, Republic of Korea; ^3^ Department of Surgery, Seoul National University College of Medicine, Seoul, Republic of Korea; ^4^ Department of Pediatrics, Seoul National University College of Medicine, Seoul, Republic of Korea

**Keywords:** home parenteral nutrition, intestinal failure, pediatrics, catheter-related bloodstream infection, intestinal failure-associated liver disease

## Abstract

**Background:**

Intestinal failure (IF) in pediatric patients often necessitates home parenteral nutrition (HPN) as a life-saving therapy. While global HPN programs are well-established, data specific to South Korea remain scarce. This study aimed to report the experience with pediatric HPN at a single center in Korea.

**Methods:**

This retrospective study included 40 pediatric patients receiving HPN between 2009 and 2021 at Seoul National University Children's Hospital. The following data of patients were collected: demographics, diagnoses, surgical factors, HPN protocols, complications including catheter-related bloodstream infection (CRBSI), intestinal failure-associated liver disease (IFALD), hospitalization records, and growth outcomes.

**Results:**

Forty pediatric patients with IF were followed for a median of 53.5 months. Common IF causes included chronic intestinal pseudo-obstruction syndrome (45.0%) and short bowel syndrome (22.5%). Six (15.0%) patients were weaned off HPN. Complications included CRBSI (60.0%, 0.90/1,000 catheter days) and metabolic issues such as IFALD (45.0%). Growth outcomes were suboptimal, with declining z-scores for weight and weight-for-height during the HPN period. No mortality was reported during the study period.

**Conclusion:**

This study provides insights into the experiences of pediatric HPN in a single center in Korea, with favorable survival and complication outcomes. However, low weaning rates and suboptimal growth outcomes highlight the need for systemic improvements including enhanced logistical support, legal reforms, and tailored nutritional strategies to optimize care for pediatric HPN patients in Korea.

## Introduction

Intestinal failure (IF) is defined as a condition in which the functional intestine is critically reduced to the point where intravenous supplements are required to meet the minimum needs for nutrients, water, and electrolytes necessary for proper growth and weight maintenance (1). Although rare, IF is a life-threatening condition that poses significant challenges for both acute and chronic medical management. Causes of IF include primary digestive diseases such as short bowel syndrome (SBS), motility disorders like chronic intestinal pseudo-obstruction (CIPO), congenital enteropathies (CE) resulting from genetic defects, and inflammatory bowel disease (IBD). Non-digestive disorders such as immune deficiencies and cancer can also lead to IF (2, 3).

In 1968, Wilmore and Dudrick published the first report showing that the growth and development of an infant could be sustained with parenteral nutrition (PN) alone (4). Since then, PN has been used for over 30 years to support proper growth and development of pediatric patients and premature infants (5). For children with IF, long-term or even lifelong PN is essential to provide adequate nutrition. Home parenteral nutrition (HPN) allows these patients to receive PN in a more familiar environment, such as their home, which can improve their quality of life and reduce financial burdens (6–8). However, parents or caregivers of these children must manage complex care routines, including preparing a sterile field, initiating and terminating infusions, managing lines and catheters, protecting devices during play, administering medications, and responding to pump alarms (2, 9). Additionally, they must recognize and address symptoms such as hypoglycemia, dehydration, or signs of infection, such as catheter-related bloodstream infection (CRBSI) (9, 10).

Medical experts understand the challenges faced by caregivers and often establish systems in hospitals to connect with home care teams to provide appropriate support and resources. In the U.S., to efficiently manage the growing number of HPN patients, a management system has been established through the American Society for Parenteral and Enteral Nutrition (ASPEN) (11). According to the ESPGHAN/ESPEN/ESPR/CSPEN guidelines, home parenteral nutrition is recommended as a safe and effective long-term therapy for children with intestinal failure, aiming to optimize survival, growth, and quality of life. In line with these international recommendations, we sought to evaluate our institutional experience with pediatric HPN in Korea, where data remain scarce (12). Thus, data for pediatric HPN are limited up to date (13). Therefore, this study aimed to report the experience with pediatric HPN at a single center in Korea. It reviewed indications and outcomes of our cohort, analyzed the incidence of complications, and compared outcomes to international literature.

## Materials and methods

### Patients and data collection

We retrospectively reviewed medical records of 40 pediatric patients who started HPN between January 1st, 2009 and December 31st, 2021 at Seoul National University Children's Hospital. This study was approved by the Institutional Review Board of Seoul National University Children's Hospital (Approval No. 2204-143-1319). Pediatric patients registered on the HPN list and managed by the pharmacy department were included in the study. Data collected included general demographics (such as age, sex, gestational age, birth weight), the presence of congenital anomalies, the patient's residence, age at the start of HPN, the primary diagnosis for HPN indication, and whether they received home care services. Surgical-related factors were gathered from surgical records, including details such as whether surgery was performed, the date of the first operation, the age at operation, preservation of the ileocecal valve (ICV), history of bowel resection, and creation of stoma (along with the type of stoma). Regarding HPN, we collected information on the type of HPN (compounded or commercial), regimen (days), calorie, volume, additional lipid emulsion administration, and method of multi-vitamin supplementation. HPN start date, duration, and the date and cause of HPN discontinuation were also collected. Data related to the central venous catheter, such as the type of central venous access device (CVAD), type of vessel used, complications related to catheter, metabolic complications related to PN, frequency, duration and reason of hospitalization, and growth parameters were retrospectively collected from medical records. Data for patients still receiving HPN were based on their status as of December 31st, 2021. For those who had discontinued HPN, the most recent data before discontinuation were used.

### PN training and follow-up

Our institution began providing pediatric HPN in January 2009. A home care service was launched in January 2019. The home care service was available to patients living within 30 km of the hospital. Regardless of whether they received home care service, all patients or caregivers starting HPN underwent PN training before discharge. Since 2019, HPN patients registered with the home care service have additionally received regular home visits from HPN nurses for about one month after discharge to ensure that PN administration is proceeding smoothly.

Electrolytes including phosphate, potassium, and magnesium were routinely monitored during PN initiation and follow-up to prevent refeeding syndrome. Follow-up visits to the pediatric surgeon or gastroenterologists occurred monthly or more frequently if needed. For clinically stable patients, during every visits, body weight, body composition, hydration status, energy and fluid balance, and biochemical tests were measured in accordance with the ESPEN guidelines (14). The composition and volume of the PN solution were adjusted at each visit in consultation with the nutrition support team (NST) based on the child's requirements and growth. Resting energy expenditure (REE) was calculated using the Schofield formula considering the child's age, weight, and height (15).

### Vascular access and handling

All children received PN via a CVAD. Depending on the patient's condition, a Chemoport, Hickman, or Broviac catheter was inserted. Both Broviac and Hickman catheters were tunneled catheters. All insertions were performed under general anesthesia with vascular ultrasound guidance to place the catheter into the superior vena cava. The insertion depth was confirmed using portable x-ray in the operating room. During outpatient visits, catheter function was checked and thrombosis was monitored using ultrasound every 12 months.

### PN intake

PN formulas were divided into compounded PN, commercial PN, or a combination of both. For compounded PN, glucose, amino acids, electrolytes, minerals, vitamins, and trace elements were customized for each patiens and prepared by our institution. The caregiver visited the hospital every two days to pick up the compounded PN bag due to PN stability concerns. In contrast, commercial PN allows caregivers to collect a month's supply at once. However, mixing micronutrients at home is challenging. Lipid emulsion was supplemented using commercial products.

### Outcome and complications

Key outcome variables for HPN included morbidity, growth parameters, and whether patients were weaned off PN. Morbidity was categorized into complications related to catheter or PN and hospitalization after starting HPN.

Catheter-related complications were classified into infections, vascular problems, and mechanical problems with the catheter. Their occurrence and frequency were collected. CRBSI was defined as the identification of the same pathogen in both peripheral and central line blood cultures with no other infection site (16). Cases where pathogens were identified only in central line blood culture (colonization) or infection localized to the catheter insertion site were classified as local infections. CRBSI incidence was calculated based on the number of CRBSI events per 1,000 catheter days. All pathogens identified during CRBSI episodes were collected. CVAD-related venous problems included venous thrombosis and stenosis caused by catheter insertion, as well as mechanical damage to the catheter (e.g., tearing, malposition, self-removal, and so on). In cases where re-insertion of a CVAD was difficult due to venous stenosis, balloon angioplasty was performed by radiological intervention.

PN-related complications included intestinal failure-associated liver disease (IFALD), gallbladder stones, renal stones, lactic acidosis, and micronutrient deficiencies. IFALD was defined as the presence of abnormal blood test results and/or abnormal ultrasound findings persisting for more than six months as follows: Alanine aminotransferase >1.5 times the upper limit of normal (67 IU/L), and/or Gamma-glutamyl transferase >1.5 times the upper limit of normal (2–4 months: >135 IU/L; 4 months–10 years: >48 IU/L; over 10 years: >36 IU/L), and/or direct bilirubin >2 mg/dl, and/or abnormal ultrasound findings such as an echogenic liver, enlarged spleen, or gallbladder stones (17). Since our institution lacked equipment to test for D-lactic acidosis, it was defined clinically as lactic acidosis identified on blood gas analysis that required hospitalization. Micronutrient deficiency was defined as requiring hospitalization due to deficiencies in elements such as copper, zinc, manganese, and selenium.

For hospitalization after starting HPN, the total number of hospitalizations per patient and the total hospitalization days were calculated. Hospitalization reasons were classified into complications related to the main disease such as ileus or small intestinal bacterial overgrowth, catheter-related complications, modification of PN formula due to metabolic complications, and other reasons.

Growth parameters for patients under 18 years old were calculated using z-scores for weight-for-age, height-for-age, and weight-for-height based on WHO Child Growth Standards (18). For patients over 19 years old, Body mass index (BMI) was calculated.

### Statistical analysis

All statistical analyses were performed using IBM SPSS 25.0. Z-scores and BMI for growth parameters are presented as means and standard deviations, while other continuous variables are presented as medians with minimum and maximum values. Nominal variables are presented as frequencies and percentages. Growth outcomes were analyzed using a paired t-test and a *p*-value of less than 0.05 was considered statistically significant.

## Results

### Patient characteristics

During the follow-up period, data from 40 pediatric patients (22 boys and 18 girls) were collected. General characteristics of these patients are shown in Table 1. The onset age of IF, meaning the age at which the diagnosis leading to HPN was first made, was less than 12 months in 23 (57.5%) patients and more than 12 months in 17 (42.5%) patients. Of patients who started HPN, 18 (45.0%) had a chronic intestinal pseudo-obstruction syndrome (CIPOS), including CIPO, hypoganglionosis, and megacystis-microcolon-intestinal hypoperistalsis syndrome. Nine (22.5%) patients started HPN due to SBS, with the underlying causes being NEC, midgut volvulus, and total colonic aganglionosis. Among the nine SBS patients, four were classified as type I (small bowel resection with high-output enterostomy proximal to the ICV) and five as type II (ileo-colic anastomosis without ICV). CE was the underlying cause in 5 (12.5%) patients, which included tufting enteropathy, diarrhea syndrome, and microvillus inclusion disease. IBD was the underlying cause in 4 (10.0%) patients, including Crohn's disease and ulcerative colitis. Additionally, non-digestive diseases leading to IF, such as hypoxic ischemic encephalopathy, cancer, and avoidant/restrictive food intake disorder, were underlying causes in 4 (10.0%) patients. A total of 31 patients underwent abdominal surgery, including 23 (57.5%) who underwent bowel resection and stoma formation and 7 (17.5%) who underwent stoma formation only. In 32 (80.0%) patients, ICV was preserved. Some patients had multiple types of stomas, with jejunostomy, ileostomy, and colostomy formed in 18, 25, and 5 patients, respectively. As the home care nursing service was initiated at our center in 2019, only 13 (32.5%) patients benefited from these services during the follow-up period. The median follow-up period was 53.5 (range, 0–155) months.

**Table 1:** General characteristics.

Parameters	*n* = 40
Gestational age (weeks)	39.0 (27.0–41.1)
Birth weight (kg)	3.20 (1.03–4.50)
Gender	
Male	22 (55.0)
Female	18 (45.0)
Onset age of intestinal failure (month)^a^	7.5 (0–235)
< 12 month	23 (57.5)
≥ 12 month	17 (42.5)
Comorbidity^b^	9 (22.5)
Cardiovascular	4 (10.0)
Chromosomal	1 (2.5)
Genitourinary	3 (7.5)
Neuromuscular	1 (2.5)
Diagnosis	
Chronic intestinal pseudo-obstruction syndrome^c^	18 (45.0)
Short bowel syndrome^d^	9 (22.5)
Chronic enteropathy^e^	5 (12.5)
Inflammatory bowel disease^f^	4 (10.0)
Others^g^	4 (10.0)
History of abdominal surgery	
Bowel resection only	1 (2.5)
Bowel resection and stoma formation	23 (57.5)
Stoma formation only	7 (17.5)
No	9 (22.5)
Ileocecal valve preserved	32 (80.0)
Type of stoma	
Jejunostomy	18 (45.0)
Ileostomy	25 (62.5)
Colostomy	5 (12.5)
Distance from patient home to center	
Seoul	18 (45.0)
Metropolitan area	12 (30.0)
Province	10 (25.0)
Home care nursing service	13 (32.5)
Follow up period (month)	53.5 (0–155)

Data is presented as the median (min—max) or *n* (%).

^a^ The age at which the diagnosis leading to home parenteral nutrition.

^b^ Diagnoses associated with home PN were excluded.

^c^ Chronic intestinal pseudo-obstruction 10 patients (25.0%), Hypoganglionosis 4 (10.0), megacystis-microcolon-intestinal hypoperistalsis syndrome 4 (10.0).

^d^ Total colon aganglionosis 4 (10.0), NEC 3 (7.5), Midgut volvulus 2 (5.0).

^e^ Tufting enteropathy 2 (5.0), diarrhea syndrome 2 (5.0), microvillus inclusion disease 1 (2.5).

^f^ Crohn's disease 3 (7.5), Ulcerative colitis 1 (2.5).

^g^ Hypoxic-ischemic encephalopathy 2 (5.0), cancer 1 (2.5), origin unknown 1 (2.5).

### PN-related characteristics

Table 2 shows PN-related characteristics. The median age of HPN beginning was 21.5 (range, 6–238) months old. HPN duration was 53.5 (range, 0–155) months. Compounded PN prepared by our center's pharmacy and commercial PN were each used by 16 (40.0%) patients, while 8 (20.0%) patients alternated between using compounded PN and commercial PN. Cyclic PN was used for most patients, with a median PN-free interval of 12 (range, 0–15) hours daily. Six (15.0%) patients were weaned off HPN. The median PN duration for these patients was 24 (range, 9–148) months. The most commonly used type of CVAD was a Chemoport in 22 (55.0%) patients, followed by Broviac catheters in 14 (35.0%) patients and Hickman catheters in 4 (10.0%) patients. The median number of CVAD insertions per patient was 4 (range, 1–15) times. The internal jugular vein (IJV) was the most commonly used site for CVAD insertion. However, in cases where insertion in the IJV was difficult due to infection, stenosis, or thrombus, the subclavian vein, external jugular vein, or brachiocephalic vein was used for CVAD insertion.

**Table 2:** Parenteral nutrition-related characteristics.

Parameters	*n* = 40
Age of HPN start (month)	21.5 (6–238)
HPN duration (month)	53.5 (0–155)
PN type	
Compounded PN	16 (40.0)
Commercial PN	16 (40.0)
Compounded + Commercial PN	8 (20.0)
Lipid emulsion	
No parenteral lipid supplementation	1 (2.5)
Composite fish oil-based	38 (95.0)
Olive oil-based	1 (2.5)
Vitamin supplement	
Parenteral	36 (90.0)
Oral intake	4 (10.0)
Micronutrients supplement	
Copper	5 (12.5)
Zinc	9 (22.5)
Selenium	30 (75.0)
PN calorie (%/REE)	76.5 (29–132)
PN volume (ml/day)	720 (240–2,152)
PN-free interval (hr/day)	12 (0–15)
Weaning off PN	6 (15.0)
Duration of patients with HPN weaned off (month)	24 (9–148)
Type of CVAD	
Chemoport	22 (55.0)
Broviac catheter	14 (35.0)
Hickman catheter	4 (10.0)
Number of CVAD insertion operation	4 (1–15)
Type of vessels used	* No. (%) of total CVC insertion (n *=* *191)*
Internal jugular vein (Rt./Lt.)	87 (45.5)/61 (31.9)
Subclavian vein (Rt./Lt.)	19 (9.9)/12 (6.3)
External jugular vein (Rt./Lt.)	4 (2.1)/5 (2.6)
Brachiocephalic vein (Rt./Lt.)	1 (0.5)/2 (1.0)

PN, parenteral nutrition; HPN, home parenteral nutrition; REE, resting energy expenditure; CVAD, central venous access device. Data are presented as median (min—max) or *n* (%).

*Indicates that the denominator used for this variable differs from the total study population.

### PN-related complications

Table 3 shows complications related to PN. Twenty-four (60.0%) patients experienced CRBSI at least once, with an median incidence of 0.90 (range, 0.33–35.53) per 1,000 catheter days. Two (5.0%) patients underwent vegetation removal surgery due to infective endocarditis caused by CRBSI. Among the total CRBSI episodes, the most common pathogens were coagulase-negative staphylococci (CoNS) with 36 (33.0%) episodes, followed by Klebsiella with 17 (15.6%) episodes, and Staphylococcus aureus with 15 episodes (13.8%). The overall incidence of CVAD-related venous problems was 0.00 per 1,000 catheter-days (median; range, 0.00–333.33), whereas the mean incidence was 8.98 per 1,000 catheter-days. Catheter-related thrombosis was identified in 9 (22.5%) patients. Venous stenosis was observed in 7 patients, among whom 3 (7.5%) patients underwent balloon angioplasty via radiologic intervention due to severe stenosis at the cavo-atrial junction.

**Table 3:** Parenteral nutrition-related complications.

PN-related complications	*n* = 40
Catheter-related complications	
Infection	28 (70.0)
CRBSI	24 (60.0)
Vegetation removal	2 (5.0)
Local infection	10 (25.0)
CRBSI incidence (/1,000 catheter days)	0.90 (0.33–35.53)
Pathogens causing CRBSI	* No. (%) of pathogens (n *=* *109)*
Gram-positive organisms	59 (54.1)
Coagulase-negative staphylococci	36 (33.0)
Staphylococcus aureus	15 (13.8)
Others	8 (7.3)
Gram-negative organisms	42 (38.5)
Klebsiella species	17 (15.6)
Escherichia coli	8 (7.3)
Enterobacter species	7 (6.4)
Acinetobacter species	4 (3.7)
Others	6 (5.5)
Fungus	8 (7.3)
Candida albicans	2 (1.8)
Candida parapsilosis	3 (2.8)
Others	3 (2.8)
CVAD-related venous problem	
Incidence (/1,000 catheter days)	0.00 (0.00–333.33)
Catheter related thrombosis	9 (22.5)
Venous stenosis	7 (17.5)
Balloon angioplasty	3 (7.5)
Catheter tearing	4 (10.0)
Catheter self-removal	2 (5.0)
Malposition	1 (2.5)
Others	5 (12.5)
PN-related metabolic complications	
IFALD	18 (45.0)
Gallbladder stone	10 (25.0)
Cholecystectomy	7 (17.5)
Splenomegaly	4 (10.0)
Pure fish oil-based lipid emulsion (Omegaven®)	2 (5.0)
Renal stone	5 (12.5)
Lactic acidosis	4 (10.0)
Micronutrient deficiency	4 (10.0)
Hospitalization during HPN	35 (87.5)
Duration of hospitalization per patient (days)	260 (13–2,070)
Number of hospitalization per patient	14 (1–49)
Cause of hospitalization	* No. (%) of total hospitalization (n *=* *501)*
Main disease	261 (52.1)
Catheter-related complications	158 (31.5)
Modification of PN formula	42 (8.4)
Others	40 (8.0)
Mortality	0 (0.0)

Data are presented as median (min—max) or *n* (%).

PN, parenteral nutrition; CRBSI; catheter-related bloodstream infection; CVAD, central venous access device; IFALD, intestinal failure associated liver disease; HPN, home parenteral nutrition.

*Indicates that the denominator used for this variable differs from the total study population.

Among PN-related metabolic complications, 18 (45.0%) patients were diagnosed with IFALD. Among them, 2 used pure fish oil-based lipid emulsion for a short period (less than 6 weeks). Gallbladder stones were confirmed in 10 (25.0%) patients, of whom 7 underwent cholecystectomy. Four (10.0%) patients had persistent splenomegaly on ultrasound for over six months. Five (12.5%) patients were diagnosed with renal stone. No cases of refeeding syndrome were observed during the study period. A total of 35 (87.5%) patients were readmitted after starting HPN, with a median duration of 260 (range, 13–2070) days and a median of 14 (range, 1–49) readmissions. Most hospitalization episodes were due to the main disease (261 episodes, 52.1%) or catheter-related complications (158 episodes, 31.5%). No deaths occurred in the study cohort.

### Growth outcomes

Table 4 presents growth parameters of patients at the time of diagnosis of the underlying causes that indicated HPN, at the initiation of HPN, and the final follow-up. The analysis of growth parameters over the HPN treatment period revealed a trend toward declining growth outcomes. The mean height-for-age z-score did not show a significant change from diagnosis −1.00 ± 2.45 to HPN initiation −1.29 ± 2.23 (*p* = 0.595), although it further declined at the final follow-up −1.86 ± 1.41 (*p* = 0.072). For weight-for-age, the z-score showed a non-significant improvement from diagnosis (−1.72 ± 2.45) to HPN initiation (−1.39 ± 2.11, *p* = 0.450), but significantly decreased by the final follow-up (−2.32 ± 1.78, *p* = 0.009). Weight-for-height z-scores initially improved from diagnosis at −1.84 ± 2.47 to HPN initiation at −0.78 ± 2.08 (*p* = 0.015), but declined at the final follow-up at −1.53 ± 1.64 (*p* = 0.049). BMI significantly improved from diagnosis (14.2 ± 2.5) to HPN initiation (16.7 ± 3.9, *p* < 0.001), but did not show a significant change from initiation to final follow-up at 15.6 ± 2.2 (*p* = 0.103). These findings indicate that, although some early improvements were observed, growth outcomes deteriorated over time, particularly in weight-for-age and weight-for-height.

**Table 4:** Clinical outcomes focusing on growth parameters.

Growth parameters	Height-for-age z-score	Weight-for-age z-score	Weight-for-height z-score	BMI (kg/m^2^)
At diagnosis	−1.00 ± 2.45	−1.72 ± 2.45	−1.84 ± 2.47	14.2 ± 2.5
At HPN start	−1.29 ± 2.23	−1.39 ± 2.11	−0.78 ± 2.08	16.7 ± 3.9
At final	−1.86 ± 1.41	−2.32 ± 1.78	−1.53 ± 1.64	15.6 ± 2.2

BMI, body mass index; HPN, home parenteral nutrition.

### Characteristics of patients with HPN weaned off

Table 5 shows six patients who were successfully weaned off HPN by December 2021. Of these six, three patients were SBS, while the remaining included one patient each with CE, IBD, and hypoxic ischemic encephalopathy. The median duration of HPN for those who succeeded in weaning was 24 months. Three of the six patients had a history of bowel resection. The ICV was preserved in four patients. Four patients could be weaned off HPN as they tolerated oral feeding well. One patient was weaned off after a serial transverse enteroplasty procedure. Another successfully discontinued PN following multi-visceral transplantation.

**Table 5:** Demographics of patients with home parenteral nutrition weaned off.

Patient no.	1	2	3	4	5	6
Gender/Age	M/17Y	M/22Y	M/6M	F/2Y	F/3Y	F/10Y
Diagnosis	CE (Tufting enteropathy)	IBD (UC)	Others (HIE)	SBS (NEC)	SBS (Midgut volvulus)	SBS (TCA)
Age at HPN start (month)	55	226	54	7	9	16
HPN duration (month)	148	31	9	12	27	21
Total follow up period (month)	155	49	22	16	27	29
History of bowel resection	No	Yes	No	Yes	Yes	No
Remnant small bowel length	N/A	>300cm	N/A	20cm	40cm	175cm
Ileocecal valve saved	Yes	No	Yes	Yes	No	Yes
Cause of PN weaning	Tolerable feeding	Tolerable feeding	Tolerable feeding	Tolerable feeding	after STEP	after TPL^a^
Weight for height z-score						
At diagnosis	−6.63		−8.63	−1.96	−0.86	3.4
At HPN start	−0.08		−5.05	−5.43	−0.2	−1.76
At final	−1.91		−0.3	−0.45	0.07	−2.47
BMI						
At diagnosis	10.01	18.27				
At HPN start	15.36	17.84				
At final	17.25	21.61				

HPN, home parenteral nutrition; BMI, body mass index; CE, chronic enteropathy; IBD, inflammatory bowel disease; UC, ulcerative colitis; HIE, hypoxic ischemic encephalopathy; SBS, short bowel syndrome; NEC, necrotizing enterocolitis; TCA, total colonic aganglionosis; STEP, serial transverse enteroplasty procedure; TPL, transplantation.

^a^ Multi-visceral transplantation (liver, small bowel and pancreas, cadaveric donor) was done.

## Discussion

IF is the reduction of gut function requiring intravenous supplementation. In pediatric patients, IF often results from critical illness, making long-term PN a life-changing necessity (1, 2). In these cases, HPN becomes a lifesaving therapy. It is the best alternative to prolonged hospitalization, enhancing the quality of life, particularly for pediatric patients (1, 12). The number of HPN patients is rising globally. To manage this increasing population effectively, the United States has established a management system through ASPEN (11). To the best of our knowledge, there are few reports in the literature regarding the prevalence and outcomes of pediatric HPN programs in South Korea (19). Our center has established an intestinal rehabilitation team based on the NST from an early stage, managing children requiring HPN through a multidisciplinary approach. Although there are still several limitations, we are one of the few large centers in South Korea implementing personalized HPN to reduce PN-related complications. Therefore, we aim to report characteristics and outcomes of 40 children enrolled in a single-center South Korea HPN cohort over a 13-year period.

In this study, the primary indication for HPN was digestive disease, accounting for 90% of cases. Notably, unlike in other studies, CIPOS accounted for 45% of these cases, followed by SBS. Non-digestive diseases such as malignancies accounted for only 10% of cases. In general, underlying diagnoses in IF patients can impact mortality rates and cause death. Particularly, non-digestive causes and motility disorders such as CIPO are known to be associated with higher mortality rates and poorer outcomes (20, 21). However, it is encouraging that, despite a high proportion of motility disorders in this study, no patients experienced mortality during follow-up.

Nevertheless, only 15% of patients achieved PN weaning, which was comparatively low given that the weaning rate in pediatric HPN patients in other studies ranged from 48% to 66% (22–24). Generally, preservation of ICV is known to be a positive prognostic factor for enteral autonomy (3, 25, 26). Although 32 (80%) patients in this cohort had preserved ICV, in the majority of these cases (21 patients), high-output jejunostomy or ileostomy was present proximal to the valve, likely rendering it functionally ineffective, which could explain the low PN weaning rate. Furthermore, previous studies have reported that pediatric CIPO patients often require long-term HPN and face significant challenges in achieving PN weaning (27, 28). The high prevalence of CIPO in this study population might have been a major factor that limited PN weaning, given the severe dysmotility associated with the disease, which could make enteral autonomy difficult to achieve.

Frequent hospital admissions due to complications can significantly impact the quality of life for both children and their families. It also increases healthcare costs (11, 22). During the total follow-up period, patients experienced an average of 14 admissions each, similar to reports from other countries regarding pediatric HPN (11, 22). Approximately half of readmissions were related to the underlying main disease, while the remainders were most commonly due to catheter-related complications.

In this study, the incidence of CRBSI was 0.90 per 1,000 catheter days, which was relatively low compared to other studies (3, 22, 29). This might be attributed to the exclusive use of tunneled catheters or implanted ports in our center. Additionally, our center uses one-lumen tunneled catheters unless there is a specific reason to use a different type. The ESPGHAN/ESPEN/ESPR/CSPEN guidelines also emphasize the importance of single-lumen tunneled catheters and multidisciplinary monitoring to minimize CRBSI risk (16, 30, 31). For home care of catheters, the use of ethanol or taurolidine locks is recommended (29, 32). However, in Korea, these agents are not available for CVAD use, making their application difficult.

The most common pathogen among total occurrences of CRBSI was CoNS, consistent with findings from various other studies (3, 33, 34). CoNS is known for its biofilm affinity, often maintaining colonization even after blood cultures have turned negative following appropriate antimicrobial treatment (35). Klebsiella species were the most frequently identified Gram-negative pathogens. This might be attributable to bacterial translocation from altered gut microbiota of patients, particularly in patients receiving long-term PN, as they are known to increase intestinal permeability (36, 37). The prevalence of fungal infections in our patient group was low, consistent with other studies. In cases of fungal infection, catheter removal is recommended. However, this can pose a significant burden for pediatric HPN patients, underscoring the need for careful management (16).

In this study, a total 9 (22.5%) patients experienced catheter-related thrombosis, of which 2 received anticoagulation, while the remaining 7 patients underwent careful catheter removal and re-insertion. Our center has educated caregivers to use heparin locks for CVAD occlusion prophylaxis. However, following a report in 2020 recommending the use of sodium chloride 0.9% instead of heparin for locking, we have since been educating caregivers to use saline locks (14).

In our study, the prevalence of IFALD was approximately 45%, higher than the 20%–23% reported in other pediatric HPN studies (3, 22, 38). This discrepancy might have stemmed from the lack of standardized definitions, particularly in pediatric cases where liver biopsies are rarely performed (39). Our study applied broader diagnostic criteria beyond bilirubin levels alone, which might have contributed to the increased prevalence. Notably, when assessed solely based on bilirubin levels, cholestasis was observed in only 10% of patients. This favorable outcome was likely influenced by the use of composite fish oil-based intravenous lipid emulsions (ILE) and the low incidence of CRBSIs (40–44). Encouragingly, all patients with IFALD recovered except for one who required multi-visceral transplantation. A pure fish oil-based ILE was used for a short period (less than 6 weeks) in 2 patients. After improvement of bilirubin levels, they were switched back to their original lipid emulsion. While risk factors such as age, prematurity, underlying diagnoses, CRBSIs, and enteral feeding have been associated with IFALD, some studies have suggested no correlation between PN duration and IFALD development (19, 45). However, IFALD was significantly related to final anthropometric outcomes, emphasizing the need for optimized nutritional management (19).

Our study highlights difficulties pediatric patients on HPN face in achieving sustained growth. Some studies have reported improved growth outcomes such as improvements in weight and height z-scores (46, 47). However, our cohort did not achieve such long-term growth stability. A critical factor affecting growth outcomes is the adequacy of nutrition. Previous studies have suggested that a parenteral nutrition dependency index (PNDI) of ≥1.5 and sufficient energy supply are crucial for normal growth (3, 34, 48). Although our study did not collect PNDI, the mean PN calorie rate was observed to be 76.5% (range: 29%–132%) of REE. This low PN calorie may reflect both gradual PN reduction during the weaning process and insufficient or inconsistent enteral intake, which could not be quantitatively assessed in our retrospective cohort. Although an important goal of HPN is to wean off PN support, our results emphasize that achieving optimal growth remains a challenge. Individualized nutritional strategies are needed to address this issue.

To establish an effective home care support system for HPN children, legal and operational improvements are needed to enhance accessibility and convenience. In South Korea, pediatric-specific commercial PN is difficult to import. Is not widely used due to insurance limitations. Adult commercial PN, on the other hand, often has high lipid levels relative to the volume needed for pediatric patients. Direct mixing of micronutrients at home by caregivers rather than by healthcare professionals is not recommended due to the risk of handling errors and associated legal concerns. Therefore, it is only used for patients whose micronutrient and vitamin levels are stable and for those whose age is closer to adulthood. This situation necessitates the use of compounded PN, which requires caregivers to visit the hospital every other day to collect the PN, causing significant inconvenience. Consequently, caregivers have expressed a preference for receiving HPN via parcel delivery services. However, due to concerns over HPN stability and legal restrictions, implementing such a system has proven challenging. Although our center started a home care nursing service in 2019, the high number of patients and the shortage of skilled personnel made delivering HPN through home care nurses challenging. In contrast, other countries have long operated systems where HPN is distributed through regional affiliated hospitals near the patient's residence, allowing periodic visits to the original center to assess the appropriateness of HPN or by outsourcing compounding and delivery services. In Korea, it is necessary to improve legal frameworks to establish similar delivery systems and hospital-affiliated networks. Overall, our results underscore both the applicability and the challenges of implementing the ESPGHAN/ESPEN/ESPR/CSPEN guideline recommendations in the Korean context, where systemic and logistical barriers remain.

Although this study provides valuable insights into the management of pediatric HPN in Korea, it has several limitations. Firstly, the retrospective design and single-center nature of the study limited the generalizability of our findings. Furthermore, precise evaluation of enteral nutrition intake was not feasible due to the retrospective nature of the study, which precluded adjustment of growth outcomes for enteral caloric intake. Secondly, the relatively small sample size and the variability in the underlying diseases and treatment durations might have influenced the observed outcomes. Thirdly, the present study did not assess quality of life for patients on HPN or compare HPN with intestinal transplantation, although these are important criteria for supporting long-term PN.

In conclusion, this study provides valuable insights into pediatric HPN in a single center in Korea, highlighting its critical role in managing IF without causing mortality. Despite low PN weaning rates and growth outcomes, effective CRBSI prevention were achieved. Challenges remain in supporting growth and improving accessibility to HPN services. To optimize outcomes for pediatric HPN in Korea, systemic legal and logistical reforms, alongside further research, are essential.

## Data Availability

The original contributions presented in the study are included in the article/Supplementary Material, further inquiries can be directed to the corresponding author.
